# Multifaceted roles of mitochondria in wound healing and chronic wound pathogenesis

**DOI:** 10.3389/fcell.2023.1252318

**Published:** 2023-09-11

**Authors:** Matthew Hunt, Monica Torres, Etty Bachar-Wikström, Jakob D. Wikström

**Affiliations:** ^1^ Dermatology and Venerology Division, Department of Medicine (Solna), Karolinska Institutet, Stockholm, Sweden; ^2^ Dermato-Venereology Clinic, Karolinska University Hospital, Stockholm, Sweden

**Keywords:** mitochondria, metabolism, wound healing, chronic wounds, hypoxia

## Abstract

Mitochondria are intracellular organelles that play a critical role in numerous cellular processes including the regulation of metabolism, cellular stress response, and cell fate. Mitochondria themselves are subject to well-orchestrated regulation in order to maintain organelle and cellular homeostasis. Wound healing is a multifactorial process that involves the stringent regulation of several cell types and cellular processes. In the event of dysregulated wound healing, hard-to-heal chronic wounds form and can place a significant burden on healthcare systems. Importantly, treatment options remain limited owing to the multifactorial nature of chronic wound pathogenesis. One area that has received more attention in recent years is the role of mitochondria in wound healing. With regards to this, current literature has demonstrated an important role for mitochondria in several areas of wound healing and chronic wound pathogenesis including metabolism, apoptosis, and redox signalling. Additionally, the influence of mitochondrial dynamics and mitophagy has also been investigated. However, few studies have utilised patient tissue when studying mitochondria in wound healing, instead using various animal models. In this review we dissect the current knowledge of the role of mitochondria in wound healing and discuss how future research can potentially aid in the progression of wound healing research.

## 1 Introduction

Wound healing is a complex and conserved process consisting of four concurrent and overlapping phases: 1) haemostasis, 2) immune response/inflammation, 3) proliferation, and 4) tissue remodelling (Velnar and Bailey, 2009), with the end goal to restore tissue integrity and homeostasis. In the event of disruption in any of four phases individually or collectively, this highly regulated process becomes dysfunctional and can subsequently lead to the formation of chronic wounds (Falanga et al., 2022). These chronic wounds are defined as a wound that does not heal within 3 months, does not heal in the orderly phases of wound healing, or does not heal after 4 weeks of treatment (Atkin et al., 2019). Chronic wounds place an enormous burden on the healthcare system, accounting for approximately 2%–4% of healthcare budgets worldwide (Olsson et al., 2019). Therefore, a greater understanding of the pathogenesis of chronic wounds and dysfunction in the wound healing process that underpins them are essential in order to improve their treatment. Additionally, due to the fact that the wound healing cycle shares similarities to healing in other tissues such as muscle (three stages of inflammation, proliferation, and remodelling) (Ciciliot and Schiaffino, 2010), liver (three stages of priming, proliferation, and termination) (Tao et al., 2017), and bone (four stages of hematoma, fibrocartilaginous callus and bony callus, as well as remodelling) (Ghiasi et al., 2017), it could be hypothesised that a better understanding of wound healing pathophysiology may also benefit research in other diseases.

Wound healing is a highly metabolically demanding process, and mitochondria play a significant role in the wound healing cycle. Mitochondria are organelles that exist in large numbers within eukaryotic cells, having originated as α-proteobacterium which became incorporated into an archaeal cell via endocytosis nearly 2 billion years ago (Sicheritz-Pontén and Andersson, 1998; Margulis et al., 2006). Human mitochondria contain their own double-stranded genome (mtDNA) roughly 16.5 kbp in size, which encodes 13 polypeptides, 22 transfer RNAs (tRNA), and two ribosomal RNAs (rRNAs). The 13 polypeptides encoded by mtDNA collectively make up a portion of the subunits of the electron transport chain (ETC). In addition to mtDNA being essential for the function of mitochondria, roughly 1,500 nuclear-encoded (nDNA) polypeptides contribute to the mitochondrial proteome (Pfanner et al., 2019). These include mtDNA and RNA polymerases, transcription factors, ribosomal proteins, and enzymes for the TCA cycle and nucleic acids, among others (Pfanner et al., 2019; Stewart and Chinnery, 2021).

Mitochondria are well known as being the cellular hubs for the generation and storage of energy in the form of adenosine triphosphate (ATP) (Spinelli and Haigis, 2018), however they are also responsible for several other key cellular processes including stress response, immunity, redox state, calcium (Ca^2+^) homeostasis, and cell fate (Shen et al., 2022). In all cases, communication between mitochondria and the nucleus is essential, in what is known as mito-nuclear crosstalk or retrograde signalling (Cardamone et al., 2018; Kim et al., 2018). In this important feedback mechanism, the mitochondria sense stress signals and relay them to the nucleus, whereby the nucleus responds by activating one or more stress response pathways (Picard and Shirihai, 2022). This can lead to alterations in mitochondrial structure and function, allowing mitochondria to adapt to what is required in order to maintain mitochondrial homeostasis. One important aspect of this is changes to mitochondrial shape (mitochondrial dynamics) and number (mitochondrial biogenesis and mitophagy) (Ma et al., 2020). In the context of wound healing, all these cellular processes are important for normal functioning, as is highlighted by a range of literature in the field and reports of mitochondrial involvement in various skin pathologies with wound healing-related characteristics (Table 1). However, in this review, we will focus on what is known about the role of mitochondria in the regulation of metabolism in wound healing, redox signalling, mitochondrial control of apoptosis, and the underlying regulation of mitochondrial homeostasis through mitochondrial dynamics and mitophagy.

**TABLE 1:** Skin pathologies with wound healing related defects and mitochondrial involvement.

Disease	Mutation	Skin defects	Mitochondrial defects	Ref
Aicardi-Goutiéres syndrome	SAMHD1	Photosensitivity; dry, scaly skin; dermatitis	Complex I deficiency; mtDNA replication defects; increased ROS	Barnérias et al. (2006), Rice et al. (2009), Dale et al. (2010), Leshinsky-Silver et al. (2011), Dragoni et al. (2022), Dragoni et al. (2023)
Epidermolysis bullosa simplex with muscular dystrophy	PLEC1	Blistering; mechanically fragile skin	Aberrant mitochondrial distribution in muscle; mitochondrial morphology abnormalities; impaired OXPHOS	Schröder et al. (2002), Maselli et al. (2011), Charlesworth et al. (2013), Tietzova et al. (2020), Vetter et al. (2020)
Werner syndrome	RECQL2	Subcutaneous calcification; ulceration	Increased ROS production; NAD depletion; impaired mitophagy	Labbé et al. (2012), Fang et al. (2019), Gudmundsrud et al. (2021)
Rothmund-Thomson syndrome	RECQL4	Atrophy; photosensitivity; ulcers	mtDNA functional defects	Croteau et al. (2012)
Cockayne syndrome B	ERCC6	Photosensitivity; scarring; decreased subcutaneous adipose tissue	mtDNA damage; increased ROS	Berquist et al. (2012), Scheibye-Knudsen et al. (2012), Scheibye-Knudsen et al. (2013), Chatre et al. (2015), Okur et al. (2020)
Pemphigus vulgaris	Multifactorial; mtABs	Blistering	OXPHOS defects; mtABs against at least 25 mitochondrial proteins; increased ROS; increased apoptosis	Di Zenzo et al. (2012), Kalantari-Dehaghi et al. (2013), Chernyavsky et al. (2015), Hutchison et al. (2022)
Systemic lupus erythematosus	mtABs; DNASE1; DcR3; fasL	Multisystemic disorder of connective tissue	T cells lymphocytes mitochondrial defects; ATP depletion; increased ROS; Δψm hyperpolarisation	Perl et al. (2004), Perl et al. (2012), Oaks and Perl (2014), Yang et al. (2020)
Psoriasis	Multifactorial, including IL-36RN; ATP2C1; CARD14; FLG; MGST2	Red, scaly skin	Decreased Δψm; elevated oxidative stress; increased apoptosis	Gabr and Al-Ghadir (2012), Sabour Alaoui et al. (2012), Mizuguchi et al. (2021), Villarreal-Martinez et al. (2023)
Kindler syndrome	FERMT1	Acral blisters; photosensitivity; progressive poikiloderma	Elevated oxidative stress; decreased Δψm; mitochondrial morphology abnormalities	Zapatero-Solana et al. (2014)

mtAB = mitochondrial antibody; Δψm = mitochondrial membrane potential.

## 2 The wound healing process

**FIGURE 1:**
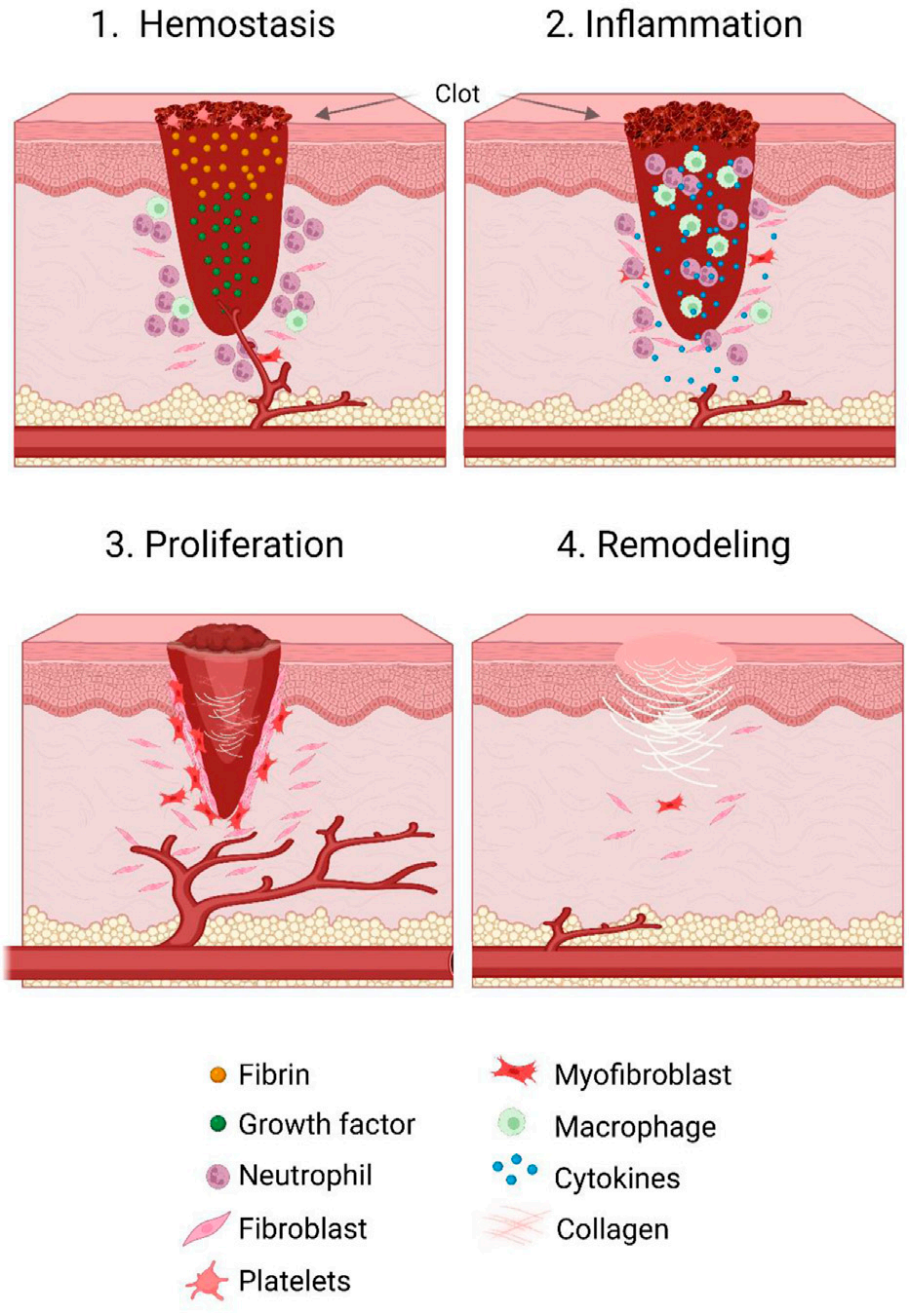
Stages of the wound healing cycle. Wound healing consists of four concurrent and overlapping stages. These include haemostasis (occurring 0 to several hours after wounding), inflammation (from 1 to 3 days post wounding), proliferation (from 4 to 21 days following wounding), and tissue repair (occurring roughly 21 days up to a year post wounding). During the haemostasis phase, inflammatory cells, growth factors and fibrin accumulate in and around the wound bed, and induce clot formation and vasoconstriction. In the inflammatory phase, several other immune cells are recruited and act as immune effector cells as well as producing growth factors. Next, in the proliferative phase, re-epithelialisation of the wound occurs, as well as formation and expansion of vasculature and granulation tissue. Finally, in the remodelling phase, contraction of the wound occurs and scar tissue is formed.

As previously mentioned, wound healing is a highly dynamic process involving the four sequential and overlapping phases of haemostasis (0-several hours following wounding), inflammation (1–3 days), proliferation (4–21 days), and tissue repair (21 days–1 year) (Reinke and Sorg, 2012) (Figure 1).

In the haemostasis phase, which occurs immediately after wounding, platelets are recruited to the wound site and aggregate, leading to clot formation and vasoconstriction. Platelets here require mitochondria for both activation and metabolic activities (Baccarelli and Byun, 2015; Melchinger et al., 2019). This subsequently induces hypoxia, with the resulting effects of pH changes and increased glycolysis (Martin, 1997; Eming et al., 2007). Following degranulation of platelets, the complement cascade is activated, leading to the stimulation of inflammatory cells and the beginning of the inflammatory phase (Sinno and Prakash, 2013). Here, in response to the hypoxic environment of the wound whereby proinflammatory mediators and damage associated molecular pattern molecules (DAMPs) are released, local immune cells are activated. Of these, leukocytic infiltration is amongst the first action (Ross and Odland, 1968). Additionally, circulating monocytes are recruited and differentiate into mature wound macrophages (Ross and Odland, 1968), as well as mast cells being recruited to the wound site from adjacent tissue (Artuc et al., 1999). In the later stages of the inflammatory phase, B and T lymphocytes are then recruited to the wound site, where they produce various cytokines and growth factors, as well as acting as immune effector cells (Barbul et al., 1989; Gillitzer and Goebeler, 2001). Leukocytes, in particular neutrophils, as well as macrophages, are responsible for clearing the wound of bacteria and pathogens. Additionally, the macrophages produce growth factors responsible for activating cells involved in the proliferation stage, such as local keratinocytes, fibroblasts, and endothelial cells, while the neutrophils are cleared by apoptosis (Ramasastry, 2005; Velnar and Bailey, 2009; Holzer-Geissler et al., 2022).

During the next phase of wound healing, proliferation, re-epithelialisation occurs. This is in addition to formation of granulation tissue and neoangiogenesis. With regards to the formation of granular tissue, collagen bundles containing fibroblasts, granulocytes, blood vessels, and macrophages create a new structure to replace the matrix that was generated during haemostasis (Schultz and Wysocki, 2009). Importantly, the primary drivers are fibroblasts. Here, fibroblasts which proliferate and migrate from the dermis produce proteinases in order to degrade the existing matrix (Hinz et al., 2007). In addition, they deposit extracellular matrix (ECM) components such as collagen, hyaluronic acid, fibronectin, and proteoglycans. These components provide a scaffold for other processes of wound healing (Barker, 2011; Xue and Jackson, 2015; Eckes et al., 2023). During re-epithelialisation, keratinocytes at the basal layer of the wound edge begin proliferating and migrating until contact, whereby new adhesion structures are formed. At this point, the keratinocytes begin to form a new basement membrane (Jacinto et al., 2001). Alongside re-epithelialisation, angiogenesis is triggered by growth factors such as vascular endothelial growth factor (VEGF). These growth factors trigger the recruitment of endothelial cells from existing vessels, where they proliferate and migrate towards the angiogenic stimulus (Li et al., 2003).

Finally, in the remodelling phase, which coincides with the end of the proliferation phase and cessation of epidermal keratinocyte migration, wound tension and contraction is driven by fibroblasts which differentiate into myofibroblasts and convert collagen III into collagen I (Hinz et al., 2007). Here, complete resolution of wound inflammation occurs, and in an attempt to recover the normal structure of tissue, granulation tissue is remodelled alongside the cell death, primarily through apoptosis, of remaining fibroblasts to form scar tissue. This new tissue contains more, and thicker, collagen than in normal tissue, and is both less vascular and less cellular than the previous tissue (Gonçalves et al., 2010; Gonzalez et al., 2016).

### 2.1 Chronic wounds–What happens when would healing goes awry?

Chronic wounds are the pathological result of partial, superficial, or full-thickness wounds failing to heal. Chronic wounds are heterogeneous with regard to several factors, such as pathophysiology, aetiology, and body location. In terms of pathogenesis, although there is no one factor that causes chronic wounds in the whole population, several factors are known to be significantly implicated. These include diabetes, neuropathies, genetic factors, arterial and venous insufficiency, certain medications, or ageing (Falanga et al., 2022). Because the wound healing process is multifactorial and highly coordinated, any disturbance in the process can lead to a failure to heal. One example where this occurs is in the inflammatory phase. Here, either a significantly enhanced inflammatory response, whereby overstimulation by immune and non-immune cells can lead to prolonged tissue inflammation (Hesketh et al., 2017; Shook et al., 2018; Januszyk et al., 2020; Kirchner et al., 2020), or an abnormal response, whereby immune cells are unable to clear invading pathogens (Falanga et al., 2022), can be common a pathophysiological cause. With regards to the tissue repair phase, dysfunction in basal cells as well as immune cells can prevent angiogenesis and re-epithelialisation (Lucas et al., 2010; Shook et al., 2016). In addition, disturbed vascularisation and angiogenesis can cause a prolonged hypoxic microenvironment which leads to excess cell death and potentially necrosis (Krisp et al., 2013). Cell proliferation and migration can also be impaired and lead to chronic wound formation due to insufficient re-epithelialisation (Makrantonaki et al., 2017a; Falanga et al., 2022).

Due to the fact that the pathophysiology of chronic wounds is so complex and multifactorial, and that effective treatment needs to be based on a multidisciplinary approach, the effort to improve knowledge in the field has proved difficult. Although several preclinical *in vivo* models of wound healing exist, such as pigs and mice, they do not display exactly the same pathophysiological mechanisms as humans (Buck et al., 2011; Dhall et al., 2014; Seaton et al., 2015; Fadini et al., 2016; Olson and Nechiporuk, 2018). A key example being that rodents predominantly heal through contraction, as opposed to re-epithelialisation (Dunn et al., 2013). Additionally, although similarities exist in invertebrate models such as *Caenorhabditis elegans* or *Drosophila*, for example, innate immune system activation, the lack of organism complexity and skin structure diversity to humans means that important processes such as angiogenesis or cell division cannot be accurately studied (Eming et al., 2014). Another point to note is that processes such as mitochondrial fragmentation or mitochondrial Ca^2+^ uptake occur at a much faster rate in these invertebrates than in humans (Ma et al., 2021). However, advancements in technologies such as improved single cell transcriptomics have allowed for more effective studies into the field, such as those studying the role of immunity (Theocharidis et al., 2022). In order to gain a more comprehensive understanding and allow for the advancement of more targeted treatments and preventions of chronic wounds, further research into specific areas of significance in the wound healing pathophysiology paradigm are needed. One such area is the role of mitochondria in wound healing. As such, in this review we will discuss in detail the current knowledge in this field and how it could be targeted in the future.

## 3 Metabolism in wound healing

Due to the complex multifactorial nature of wound healing–whereby many cellular processes such as regeneration of tissue (Rousselle et al., 2019), immune response (Hall et al., 2014; Willenborg et al., 2020), replenishment of intracellular contents and phospholipid membranes (Xu and Huang, 2020), as well as proliferation and migration of various cell types (Onida et al., 2019), require vast amounts of energy–wound healing is a highly metabolically demanding process.

### 3.1 Metabolic pathways

As previously mentioned, mitochondria are the principle suppliers of energy in the cell. They produce ATP as well as metabolic precursors. In addition, mitochondria also generate metabolic by-products essential for signalling and cellular homeostasis such as ammonia and reactive oxygen species (ROS) (Spinelli and Haigis, 2018).

The most widely known process by which mitochondria supply energy is through the metabolism of nutrients into ATP via the TCA cycle and ETC (Watt et al., 2010; Walsh et al., 2017). In addition to ATP, various other metabolites are produced by mitochondria. These include pyruvate, glutamine, branch-chain amino acids, and fatty acids (FAs). Pyruvate is produced in the cytosol via glycolysis in normoxic conditions and shuttled through the inner mitochondrial membrane (IMM) into mitochondria. However, during hypoxia, pyruvate picks up electrons and generates lactate in the cytosol (Papandreou et al., 2006). Branched-chain amino acids are sources of ATP for cells such as myocytes and adipocytes during differentiation (Green et al., 2016; Shimomura et al., 2023). FAs, such as palmitate, are significant sources of energy for cellular function, particularly in nutrient stress conditions (Rohrig and Schulze, 2016).

An alternative form of energy production in cells is through the process of aerobic glycolysis, which is advantageous for proliferating cells as well as cells in hypoxic or tumour environments where the availability of amino acids, FAs, and nucleotides is more important than that of ATP (Li et al., 2014). During glycolysis, glucose is converted into pyruvate in the cytoplasm, producing ATP (Li et al., 2014). In addition to aerobic glycolysis, glutamine is converted into glutamate, lactate, and ammonia by glutaminase (GLS) in a process termed glutaminolysis (Divakaruni et al., 2013; Schell et al., 2014). This process is critical in order to maintain the energy requirements of proliferating cells (DeBerardinis, 2007; Wise et al., 2008; Wang, 2011a).

As stated above, mitochondria also act as hubs for the generation and storage of other biometabolic molecules. One example is that mitochondria generate citrate as a TCA cycle intermediate. Citrate is important as a regulator of anaerobic respiration by acting as a carbon source (Hatzivassiliou et al., 2005) and is involved in the growth and proliferation of various cell types (Ben-Sahra et al., 2016; Ron-Harel et al., 2016).

### 3.2 Roles of metabolism in normal wound healing

**FIGURE 2:**
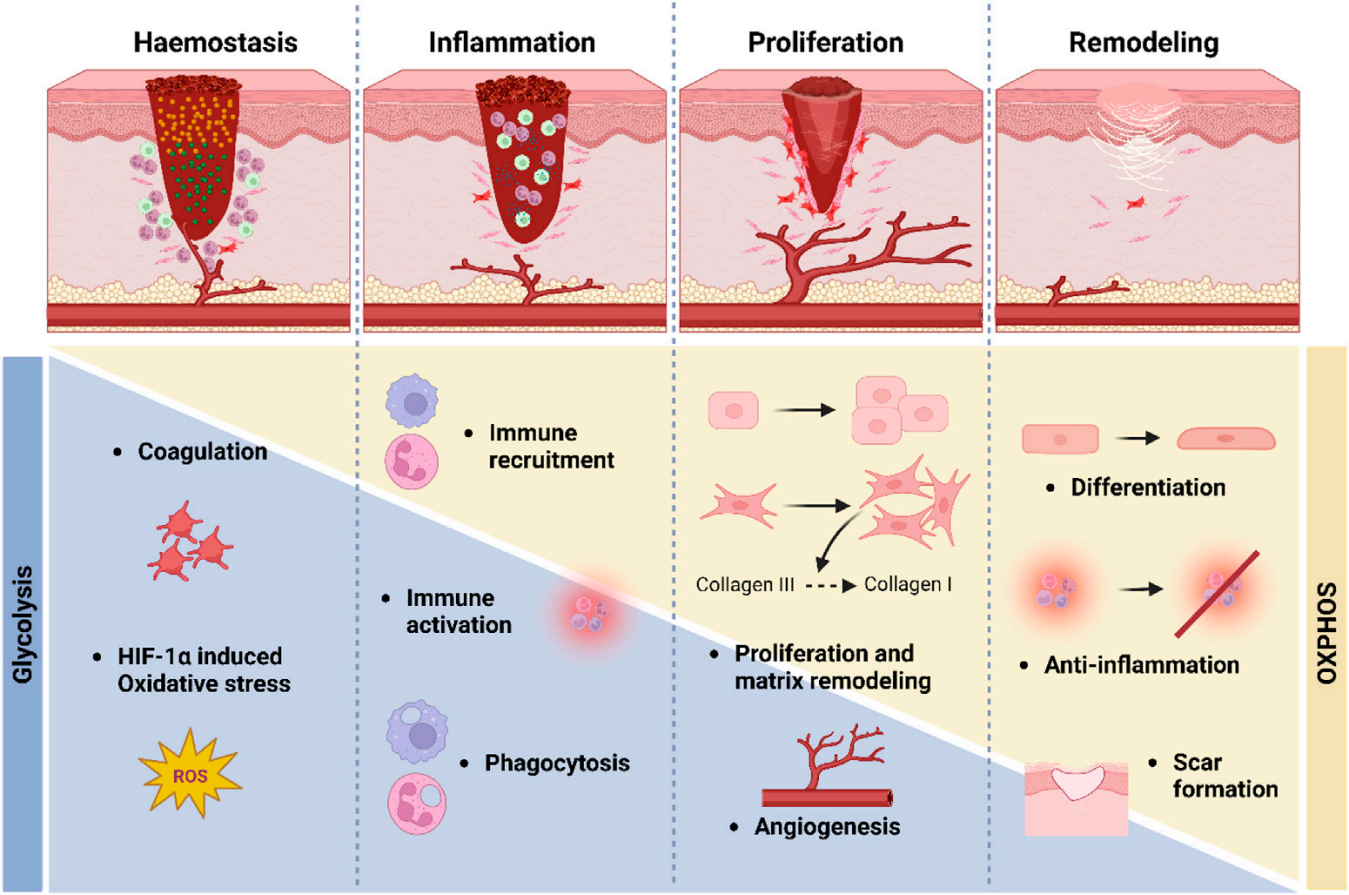
Metabolism in wound healing. During the early stages of wound healing, primarily the haemostasis and inflammation stages, glycolytic metabolism is upregulated, and there is a simultaneous downregulation of oxidative metabolism. Later, during the proliferation and remodelling stages, there is a switch from glycolytic metabolism (blue bar) towards more oxidative metabolism (yellow bar).

In general, wound healing is associated with a switch in metabolism towards anaerobic metabolism, due to the hypoxic nature of the wound milieu (Mannello et al., 2014; Eming et al., 2021) (Figure 2). A recent paper by our lab assessed the metabolic profile of wounding in human skin. In it, it was confirmed that glycolysis, and to a lesser extent glutaminolysis, are vital for the normal regulation of wound healing, and that defects in these pathways may be a pathogenic factor in chronic wound formation (Manchanda et al., 2023). In this study it was demonstrated using various techniques such as metabolomics and transcriptomics that there is an upregulation of glycolysis, glutaminolysis, the TCA cycle, and β-oxidation in both acute and chronic wound skin samples. In concert with this, levels of mtDNA, encoding oxidative phosphorylation (OXPHOS) subunits, and nDNA genes involved in fatty acid oxidation, were significantly downregulated in the wounded samples compared to intact skin (Manchanda et al., 2023). Additionally, explant work further demonstrated the significance of upregulated glycolysis and glutaminolysis in efficient wound healing and in particular, the proliferation and migration of fibroblasts and keratinocytes.

This paper has built upon previous studies which demonstrated greater glucose utilisation and lactate production in both burn patients and animal models (Im and Hoopes, 1970; Vinaik et al., 2020). In another recent study which investigated the differences of cells involved in wound healing at the single-cell level in wounded mice, the cells in the neo-epidermis 4 days post wounding exhibited a high glycolysis state, compared to unwounded cells which exhibited a low glycolysis state (Haensel et al., 2020). Accordingly, basal cells in unwounded skin showed the highest levels for OXPHOS, whereas the cells in the wounded skin had significantly lower levels of OXPHOS. Additionally, wound edge keratinocytes with increased migration and differentiation have been shown to have elevated levels of the polyamine spermine, an endogenous polyamine metabolite which increases cell proliferation and differentiation through DNA synthesis (Zhan et al., 2015; Lim et al., 2018; Ito et al., 2021). Polyamines were also found to be elevated in chronic wound samples in the recent Manchanda *et al.* study (Manchanda et al., 2023). Other studies have also demonstrated the beneficial effects of inhibiting OXPHOS on wound healing (Chen et al., 2003; Ved et al., 2005; Xu and Chisholm, 2014).

With regards to the inflammatory phase of wound healing, previous studies have demonstrated alterations in epidermal cell metabolism in the immune response (Hall et al., 2014; Willenborg et al., 2020). Recent studies have examined the differences between the two types of macrophages involved in wound healing. Here it was shown that type-1 macrophages, primarily involved in the inflammatory phase and so with antimicrobial functions and proinflammatory actions, utilised glycolysis (Eming et al., 2017; Willenborg et al., 2020). Conversely, M2 macrophages that are involved in the later phases of wound healing utilised OXPHOS. They also found that these macrophages had signatures for anabolic metabolism. Due to the distinct differences in metabolic signature between and M1 and M2 macrophages, metabolic reprogramming is of vital significance, and disturbances in this metabolic reprogramming may affect the proper transition of macrophage subtype (Mills and O'Neill, 2016). Studies have also provided evidence that there is a specific composition of lipids required in the inflammatory stage (Wijesinghe et al., 2014; Wijesinghe et al., 2016; Yasukawa et al., 2020), and so it could be hypothesised that fatty acid oxidation plays an important role in wound healing.

As with the upregulation of glycolysis required for the proinflammatory effects of the M1 macrophages involved in the inflammatory phase, these macrophages seemingly also utilise glycolysis in order to promote angiogenesis (Willenborg et al., 2020). Metabolism is also critically involved in endothelial cell function in angiogenesis (De Bock et al., 2013; Schoors et al., 2015; Kim et al., 2017; Eelen et al., 2018; Diebold et al., 2019). Here, OXPHOS, which is upregulated in the latter stages of wound healing, is required for efficient migration and proliferation of endothelial cells and therefore angiogenesis, which also occurs in the more latter stages of wound healing (Vandekeere et al., 2018; Schiffmann et al., 2020).

Another process that metabolism is important in wound healing for is in matrix remodelling. Previously, glucose treatment was found to accelerate the formation of collagen and therefore re-epithelialisation in *ex vivo* wounded skin, while collagen genes were upregulated in 2-Deoxy-D-glucose (2-DG) treated fibroblasts (Manchanda et al., 2023). In a separate study, L-glutamine treatment was shown to enhance the rate of collagen biosynthesis and re-epithelialisation in a rat incision wound model (Goswami et al., 2016). Thus, modulating metabolism may be a potential target for treating chronic wounds.

### 3.3 The role of metabolism in chronic wound pathogenesis

Due to the fact that metabolism plays such an important role in wound healing, it is of no surprise that defects in the regulation of metabolism may be involved in the pathogenesis of chronic wounds. One possible mechanism may be the long-term upregulation of lipids derived from free FAs even after the normal course of wound healing has finished, thereby prolonging inflammation in the wound (Yasukawa et al., 2020; Manchanda et al., 2023). As mentioned earlier, this is significant, as prolonged inflammation can render wound healing stuck in the inflammatory stage. Prolonged inflammation may also arise in chronic wounds by the elevated levels of polyamines (Zhan et al., 2015; Lim et al., 2018).

The reports detailed in this section offer a reasoning to the hypothesis that dysregulated metabolism may be important in the formation of chronic wounds, and therefore that methods to target this dysfunction may be a viable treatment option for chronic wounds. Some examples of this include evidence of topical insulin treatment increasing glucose availability in a type II diabetic ischemic rat wound model (Yu et al., 2017), as well as honey acting as a beneficial fuel for glycolytic metabolism in wound healing (Sumitra et al., 2009). However, in the latter example, it should also be stated that other components of honey may also contribute to the beneficial wound healing effect.

## 4 Mitochondrial regulation of ROS

Mitochondria are the primary producers of reactive oxygen species (mtROS) and of redox signalling in the cell, producing roughly 90% of ROS (Balaban et al., 2005a). As a consequence of proton transfer during OXPHOS, electrons leak from the ETC, complexes I, II, III, and IV. This results in the production of superoxide (Quinlan et al., 2013). Superoxide is then converted into hydrogen peroxide (H_2_O_2_) by superoxide dismutases. Subsequently, H_2_O_2_ is detoxified, generating water and oxygen (Shadel and Horvath, 2015). Importantly, following diffusion through the mitochondrial membranes, H_2_O_2_ that is not detoxified is then responsible for regulating redox signalling (Shadel and Horvath, 2015).

As previously mentioned, the wound milieu is hypoxic due to the disruption of vasculature around the wound and subsequent decrease in oxygen supply (Haensel et al., 2020). As such, there is a significant decrease in oxidative metabolism, and an upregulation in aerobic glycolysis (Im and Hoopes, 1970; Haensel et al., 2020). The result of this is the formation of a microenvironment whereby metabolic intermediates required for cell proliferation and migration such as lactate are more readily available (Vinaik et al., 2020). Hypoxia is known to induce the stabilisation of HIF-1α and the upregulation of mtROS (Chandel et al., 1998). Together these lead to the downstream activation of AMP-activated protein kinase (AMPK), mitogen-activated protein kinase (MAPK), and nuclear factor erythroid-derived 2-like 2 (Nrf2) pathways, which are critically involved in apoptosis (see Section 5), differentiation, motility, and proliferation of cells (Lamb et al., 2003; Lu and Xu, 2006; Kim and Choi, 2015; Atay and Skotheim, 2017). However, studies have indicated that the concentration of H_2_O_2_ in the wound is between 100 and 250 μM, and the various processes of wound healing require precise levels of ROS. As such, imbalances in the ROS equilibrium either way can be deleterious, and often lead to the formation of chronic wounds (Polaka et al., 2022).

### 4.1 mtROS in cell migration and antimicrobial functions in wound healing

**FIGURE 3:**
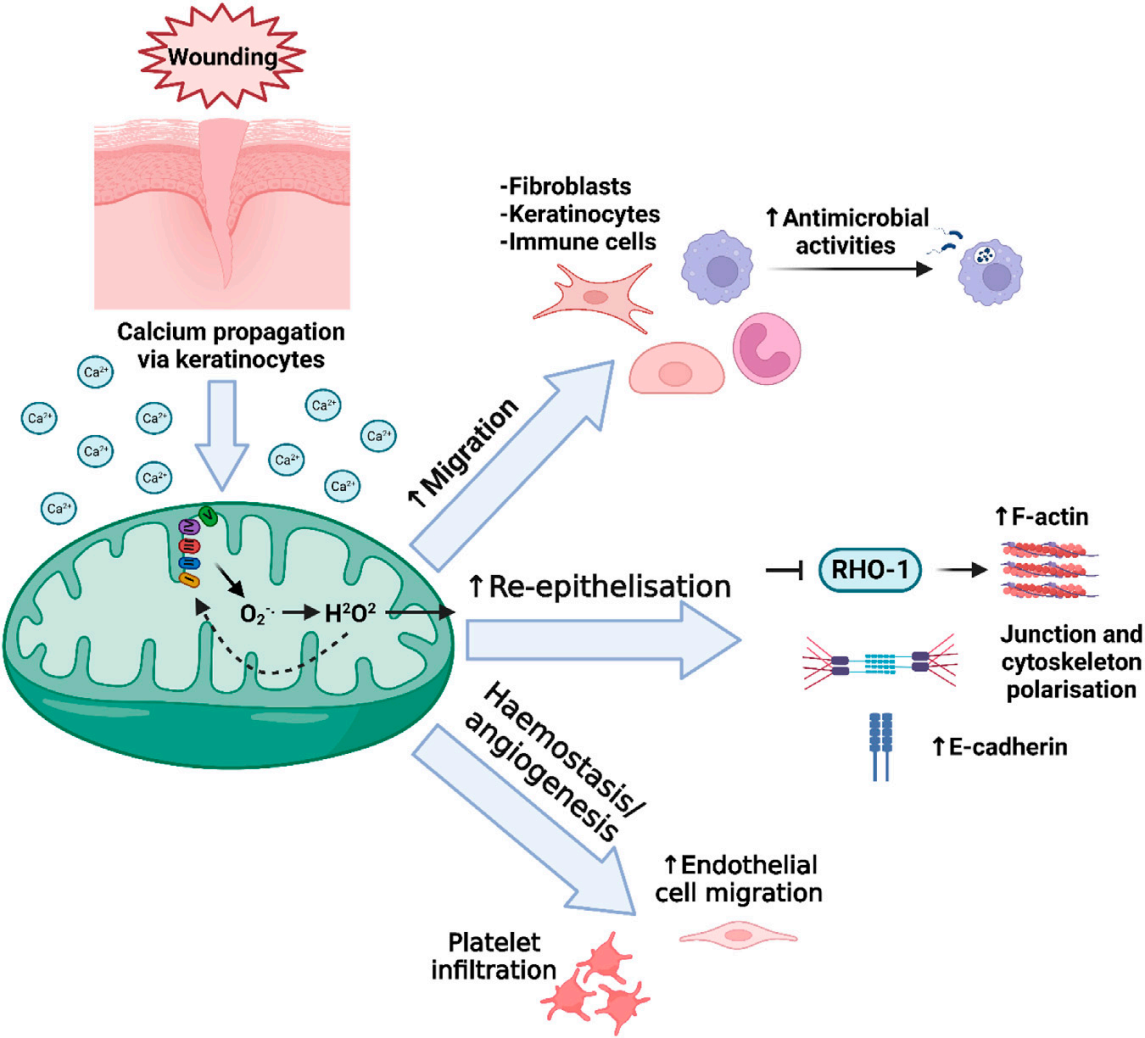
Mitochondrial redox signalling in wound healing. Following wounding, a burst of intracellular Ca^2+^ is released as the initial damage signal, and is detected by mitochondria. Among several subsequent actions, increase in mitochondrial Ca^2+^ can lead to the increased leakage of ROS from the ETC. After conversion of superoxide (O_2_^−.^) into H_2_O_2_, the H_2_O_2_ is translocated out of the mitochondria though its membrane pores including VDAC, and into the cytosol. Here, this H_2_O_2_ can have several effects that contribute to the regulation of wound healing, including involvement in the migration of immune and non-immune cells, as well as antimicrobial activities of immune cells, accelerating re-epithelialisation, and regulating platelet infiltration in the initial haemostasis phase and later, accelerating the migration of endothelial cells involved in angiogenesis.

mtROS production at the early stages of wound healing has pleiotropic effects, and is critical for the efficiency of the wound healing process (Gauron et al., 2013; Love et al., 2013) (Figure 3). Importantly though, whilst low levels of ROS are essential in wound healing, excessive production of ROS can lead to oxidative damage and impaired wound healing (Cano Sanchez et al., 2018). One beneficial example of mtROS is that of H_2_O_2_, which recruits inflammatory cells such as phagocytes to the wound site (Niethammer et al., 2009a; West et al., 2011; Yoo et al., 2011; Pase et al., 2012; Razzell et al., 2013; van der Vliet and Janssen-Heininger, 2014). In the recent Willenborg et al. paper, which profiled early and late wound stage macrophages in mice, it was confirmed that macrophages involved in the early events of wound healing have high levels of mtROS production, high expression of mtROS detoxification genes, high levels of succinate metabolism, and subsequently high HIF-1α stabilisation, coinciding with upregulated glycolysis (Willenborg et al., 2020). In addition to its role in the recruitment of immune cells in wound healing, mtROS such as H_2_O_2_ also aid in antimicrobial mechanisms of immune cells (Cao et al., 2022). mtROS also play an important role in the signalling, survival, and cytokine production of immune cells, which are essential functions required to prevent infections and therefore prolonged inflammation (Allaoui et al., 2009; Segal et al., 2012; van der Vliet and Janssen-Heininger, 2014; Dunnill et al., 2017; Hoffmann and Griffiths, 2018).

mtROS have also been shown to be important in migration and adhesion of non-immune cells during both development and wound healing in various *in vitro* models (Chiarugi et al., 2003; Kwon et al., 2016; Weidong P et al., 2022). During cell migration, cells require signals which trigger the polarisation and extension of protrusions in the direction of chemoattractants, of which H_2_O_2_ is a key one (Klyubin et al., 1996; Hurd et al., 2012). Previous studies using cell and mouse models have demonstrated the beneficial effect of hypoxia and production of mtROS on keratinocyte motility and migration via p38 and JNK/MAPK signalling during wound healing (O’Toole et al., 2008; Rodriguez et al., 2008; Zhang et al., 2019). With regards to dermal fibroblasts, hypoxic conditions significantly enhance their proliferation as well as their differentiation into myofibroblasts, as shown in previous studies on various disease rodent models and cells (Falanga and Kirsner, 1993; Sen and Roy, 2010; Janda et al., 2016; Levigne et al., 2016; Hoffmann and Griffiths, 2018).

### 4.2 mtROS and re-epithelialisation

During re-epithelialisation of skin during wound repair, several proteins act to aid in keratinocyte migration and proliferating, as well as the formation and regulation of the cytoskeleton. These include F-actin, myosin, and E-cadherin (Rousselle et al., 2019). Importantly, studies have shown that mtROS promotes wound healing by regulating F-actin and myosin at the wound edge (Xu and Chisholm, 2011; Muliyil and Narasimha, 2014; Xu and Chisholm, 2014; Hunter et al., 2018; Fung et al., 2023). Of note, in the later Xu and Chisholm study they demonstrated that wounding in *C. elegans* induced a rapid release of Ca^2+^ around the wound site and uptake into mitochondria, subsequently inducing mtROS production. This mtROS then inhibits RHO-1 activity via oxidation of cysteine residues (Lambeth and Neish, 2014) to enhance F-actin build up and accelerate wound closure (Xu and Chisholm, 2014). The more recent study by Hunter et al. confirmed the key role of mtROS in the promotion of E-cadherin trafficking as well as junction and cytoskeleton polarisation in wound closure. Again, it was shown that Ca^2+^ signals released immediately after wounding induced a greater production of mtROS, leading to cytoskeleton remodelling mediated by redox signalling (Hunter et al., 2018).

### 4.3 Vascularisation, angiogenesis and mtROS in wound healing

Angiogenic factors are released into the wound bed immediately following wounding and granulation tissue begins to form between day three and five post wounding, allowing for the formation of new blood vessels via angiogenesis, as well as fibroblast proliferation, and collagen production (Tonnesen et al., 2000). Along with NADPH oxidase (NOX), mtROS play an important role in angiogenesis in the wound healing response (Knighton et al., 1983; Guo et al., 2010; Schreml et al., 2010; Aldosari et al., 2018). In addition, mtROS-sensitive pathways mentioned previously, such as the HIF-1α, NFκB, MAPK, JAK, PI3 kinase, and Akt pathways are involved in angiogenesis in various other tissues (Chandel et al., 2000; Guzy et al., 2005; Klimova and Chandel, 2008; Fukai and Ushio-Fukai, 2020).

In the early stages of wound healing, mtROS induce the recruitment of platelets and other immune cells to the wound site (Hoffmann and Griffiths, 2018). mtROS is also significantly involved in the migration of endothelial cells in wound healing. In particular, mtROS promote endothelial cell migration as a downstream effect of VEGF (Wang et al., 2011b). In both these instances, the effects of mtROS are dependant on wounding-induced hypoxia, and although these studies show a positive correlation, in some instances it is difficult to uncouple the role of mtROS from hypoxia. Although there are few studies describing the specific role of mtROS in angiogenesis, current thinking supports the suggestion that mtROS is critical agent in angiogenesis occurring during wound healing.

### 4.4 Skin ageing, senescence, and wound healing

Wound healing is recognised to be delayed in aged skin due to a multitude of factors (Ding et al., 2021). Importantly, aged skin is structurally different to younger skin, characterised by having decreased keratinocyte turnover (Biniek, 2015), impaired cellular plasticity (Ocampo et al., 2016), decreased fibroblast density (Salzer et al., 2018), less efficient collagen maturation as well as organisation (Kobayashi et al., 2019), and thinner vasculature (Bentov and Reed, 2015). Immune responses and immune cell abundance (Nishio et al., 2008; Vu et al., 2022) as well as keratinocyte dynamics (Keyes et al., 2016) are also dysregulated in wound healing in aged skin. In addition, inefficient metabolism was found to contribute to delayed wound healing in aged mice (Jones et al., 2020), and in a separate study macrophage metabolism was found to be altered in aged wounded skin compared to young wound skin (Vu et al., 2022). Current thinking supports the idea that mitochondria are implicated in several of these age-related dysregulations in aged skin wound healing (Schiffmann et al., 2020; Sreedhar et al., 2020). Indeed, mitochondrial abnormalities are a common feature of aged skin in general (Stout and Birch-Machin, 2019; Sreedhar et al., 2020), whereby mtDNA deletions such as the ‘common mutation’ accumulate and are accompanied by impaired mitochondrial function, characterised broadly by elevated oxidative stress and loss of mitochondrial membrane potential. In particular, the role of mitochondria in metabolic dysfunction is of interest, as impaired vascularisation in aged skin occurs as a consequence of decreased HIF-1α expression (Bonham et al., 2020). Indeed, studies in aged mouse skin demonstrated that increased HIF-1α stabilization through topical deferoxamine (DFP) administration accelerated wound healing (Duscher et al., 2017). DFP is also accepted to be a mitophagy inducer (Allen et al., 2013), and so although not researched in the context of wound healing, this increased DFP-mediated vascularisation may also be related to accompanying elevated mitophagy. The most seemingly promising other results with regard to targetting mitochondrial function in aged skin as a potential treatment for wound healing is metformin, a caloric restriction mimitec. Here, metformin accelerated wound healing in aged mice through improved AMPK-mediated angiogeneisis (Zhao et al., 2017).

Cellular senescence is a state of irreversible cell-cycle arrest brought about in response to various cellular stressors, and is characterised by a pro-inflammatory phenotype termed the senescence-associated secretory phenotype (SASP). The SASP is known to increase in aged tissue, particularly that of proliferative cells, and is implicated in pathogenic defects which contribute to various physiologies, including wound healing (Gorgoulis et al., 2019; von Zglinicki et al., 2021). In recent years, mitochondria and mitochondrial dynamics have been significantly implicated in cellular senescence in various contexts (Westermann, 2010), and in particular in aged and UV-damaged skin (Sreedhar et al., 2020). Here, as the result of oxidative damage, mutations in mtDNA can accumulate and lead to aberrations in various aspects of mitochondrial features (Yang et al., 1995; Berneburg and Krutmann, 2000; Berneburg and Lehmann, 2001; Balaban et al., 2005b; Krishnan et al., 2005; Schroeder et al., 2008; Kaneko et al., 2012; Sreedhar et al., 2020) and subsequently induce cellular senescence (Martini and Passos, 2022).

Transient senescence has been shown to be important in wound healing (Wilkinson and Hardman, 2020), for example, by preventing excessive fibrosis (Krizhanovsky et al., 2008; Jun and Lau, 2017; Hiebert et al., 2018). However, chronic senescence in various cell types can propagate impaired wound healing through various mechanisms, such as prolonged inflammation, epidermal hyperproliferation, matrix proteolysis, and impaired anti-microbial activities (Swift et al., 2001; Ressler et al., 2006; Wall et al., 2008; Makrantonaki et al., 2017b; Albanesi et al., 2018; Wilkinson et al., 2019). As such, targeting uncontrolled senescence may be a potential therapeutic target for future treatments.

With regards to UV-induced damage in skin, it has been shown that UVA-induced mtDNA deletions in both fibroblasts and keratinocytes lead to the increased activity of collagen-degrading enzymes (Berneburg and Krutmann, 2000), downregulation of collagen biosynthesis genes (Blatt and Littarru, 2011), and ECM degradation (Oyewole et al., 2014). In addition to the common phenotypes of aged skin, such as impaired proliferative, migratory, and differentiation ability of various cell types (Sreedhar et al., 2020), UV-induced and aged skin-induced mitochondrial damage and senescence can lead to chronic inflammation, which is a common phenotype of chronic wound pathophysiology (D’Orazio et al., 2013).

### 4.5 Targeting mtROS as a therapeutic strategy in wound healing

As mentioned previously, mtROS play a pleiotropic role in wound healing, and persistently exist at low levels throughout the process. Precise, low-level ROS is beneficial at several adaptive stages of wound healing (Polaka et al., 2022). However, excessive ROS production can be damaging and impair wound healing. For example, at the inflammatory stage, where excessive ROS levels can cause chronic inflammation (Sen and Roy, 2010; Bryan et al., 2012). Prolonged ROS production can also enhance the activation of pro-apoptotic proteins, leading to excessive apoptosis and necrosis (Trachootham et al., 2008). In line with this, several studies have described the beneficial effects of targeting mtROS in wound healing in an effort to prevent excessive ROS production and treat chronic wounds (Kunkemoeller and Kyriakides, 2017; Cano Sanchez et al., 2018). Endogenous antioxidant compounds such as taurine, N-acetyltaurin and hypotaurine have been found in wound samples from humans (Manchanda et al., 2023). Pharmaceutical regulation of ROS has also been shown to be beneficial in wound healing in diabetic mice. For example, administration of SkQ1, which normalises ROS levels in diabetic mice, accelerated wound healing by enhancing re-epithelialisation, vascularisation, and granulation tissue formation, as well as improving immune cell regulation (Demyanenko et al., 2017). It was also found to accelerate granulation tissue formation, vascularisation and inflammatory phase resolution in aged mouse models (Demyanenko et al., 2015). Other studies investigating the impact of various antioxidants in diabetic wounds include the genetic inhibition of XO, or activation of Nrf2-mediatiated antioxidants, which significantly improved wound healing (Bitar and Al-Mulla, 2011; Weinstein et al., 2015; Soares et al., 2016; David et al., 2017). In addition to various chemicals, extensive research has gone into developing novel applications to regulate ROS levels in wounds. One such example are oxygen-releasing polymeric microspheres embedded in alginate hydrogels (Choi et al., 2018). These microspheres were found to enhance cell growth and thus wound healing, primarily through upregulating angiogenesis via increased oxygenation around the wound. In a recent review by Polaka et al., the various current treatment possibilities for modulating ROS in wound healing are described in more detail (Polaka et al., 2022).

## 5 Apoptosis in wound healing

Apoptosis is a non-inflammatory process that leads to programmed cell death and is essential for tissue homeostasis, defence, and development. It is particularly important in wound healing for aspects such as the removal of inflammatory cells in early healing and the formation of the scar (Greenhalgh, 1998). The apoptotic process is characterised by specific morphological changes. First, the cell shrinks and ceases to adhere to the neighbouring cells, albeit maintaining an intact cell membrane. Then, chromatin condensation, membrane blebbing, and nuclear fragmentation take place (Kerr et al., 1972). In the meantime, cytochrome c is released from a ruptured mitochondrial membrane and activates a series of degradation reactions that lead to the formation of apoptotic bodies. The remaining particles are quickly cleared by phagocytosis, without triggering inflammation (Taylor et al., 2008). Apoptosis is a balanced state between the action of pro- and anti-apoptotic factors. Activation can occur via an extrinsic pathway, through ligand-binding activation of death receptors (CD95/Fas, tumour necrosis factor (TNF) receptor-1) (Ashkenazi and Dixit, 1998), or via an intrinsic pathway through an intracellular cascade of reactions dependent on mitochondrial permeabilisation changes and the release of cytochrome c (Scaffidi et al., 1998). The regulation of apoptosis was initially thought to happen at the nuclear level. However, when Bcl-2 was reported to be localised in the mitochondria (Hockenbery et al., 1990), the mitochondrial permeability transition pore (mPTP) was reported to generate the “point of no return” for apoptosis (Kroemer et al., 1995), and cytochrome c was determined essential for the activation of caspases (Liu et al., 1996), mitochondria ended up at the centre of the apoptotic regulation process, as well as a means of amplification of the signal generated via the extrinsic pathway (Bossy-Wetzel and Green, 1999).

### 5.1 Apoptosis is necessary for normal wound healing

Wound healing consists of overlapping phases and the correct transition from one phase to the next, following the required timeline, is necessary for adequate healing. Apoptosis has an essential role in this context. It is necessary to remove inflammatory cells during inflammation, and the absence of this response leads to persistent inflammation and non-healing wounds. Furthermore, apoptosis balances the proliferative phase by not allowing excessive cell proliferation and by reducing the cell load on granulation tissue at a later stage, to allow scar formation. Several mice and pig studies showed the impact of apoptosis in wound healing and the impact of non-efficient apoptosis in disease models, such as in diabetes.

Brown et al. (Brown et al., 1997) demonstrated that apoptosis is involved in downregulating inflammation while the re-epithelialisation tissue is generated at the edges of the wound. They assessed wounds in diabetic and non-diabetic mice and detected (in normal mice) early apoptosis in inflammatory cells (earliest 12 h), peaking at day 5 and then regressing. Apoptosis was consistently seen in inflammatory cells underneath the edge of re-epithelialisation tissue or tongue, which suggests that apoptosis may be an important source of signalling in the transition between the inflammatory and proliferative phases. Diabetic mice in this study showed delayed apoptosis and slower healing. Further studies explored apoptosis of inflammatory cells and their role in guiding re-epithelialisation. Kane *e*t al. (Kane and Greenhalgh, 2000) compared apoptosis between diabetic and non-diabetic mice for 42 days and assessed the expression of p53 and Bcl-2. In normal mice they observed an inversed correlation between the levels of Bcl-2 and p53 over time, where Bcl-2 levels increased just after wounding (in parallel to decreasing p53) to allow cell proliferation. When the inflammation phase decreased, p53 levels raised in contrast to Bcl-2, and the rate of apoptosis seemed to be consistent with the levels of p53. This was not seen in diabetic mice, where a consistently higher expression of p53 in relation to Bcl-2 was observed. Nagata et al. (Nagata et al., 1999), in a study using pig burn wounds, also showed that apoptosis and the expression of p53 were higher during the proliferative phase, later declining during the remodelling phase. The importance of apoptosis in the maturation and evolution of granulation tissue was demonstrated by Desmouliere et al. (Desmoulière et al., 1995). In a rat model (8 weeks old), wounds were inflicted dorsally, and granulation tissue was sampled and analysed from day 2–60 (at various time points). Apoptosis was observed initially after wounding, became more evident at day 8, and peaked between day 12 and 25. During this last period, the frequency of apoptotic myofibroblasts and vascular cells was the highest, which suggests that the apoptosis of the granulation tissue occurs after wound closure and takes place in a consecutive manner, leading to a gradual clearance of the granulation tissue over time. Other aspects linked to dysregulation of apoptosis in skin wound healing were, for example, hypertrophic scars (Wassermann et al., 1998) but also decreased myofibroblast differentiation leading to delayed wound contraction (Sindrilaru et al., 2009).

### 5.2 Mitochondrial permeability transition and apoptosis

Mitochondrial permeability transition (MPT) is a state characterised by an increase of the inner membrane permeability, mediated by voltage-dependent channels located in the inner membrane such as the mPTP. The exact composition of the mPTP is still not fully understood, but it is recognised that the pore is composed of several proteins such as the adenine nucleotide translocator (ANT) and Cyclophilin D (CypD), a target for the drug Cyclosporine A, which inhibits CypD and mediates the closure of mPTP (Beutner et al., 1997). A transient loss of membrane potential in the IMM occurs in certain physiological states and leads to outcomes such as muscle contraction or saliva secretion (Altschuld et al., 1992; Ryu et al., 2010). It can also mediate cell signalling by generating a burst in ROS production (Wang et al., 2008). However, a prolonged dissipation of the inner membrane potential is usually linked to cell death (Marchetti et al., 1996; Zamzami et al., 2005).

During the intrinsic apoptotic pathway, there is a tight regulatory system in place, led by the Bcl-2 protein family (Kroemer et al., 2007). Bax and Bak are necessary for the machinery behind membrane permeabilisation in apoptosis. On the contrary, Bcl-2 and Bcl-xL act as anti-apoptotic factors. In addition, BH3-proteins can interact with both anti- and pro-apoptotic effectors (Chipuk and Green, 2008). This way, the Bcl-2 protein family has a direct effect on cell fate depending on the upstream stimulus, deciding whether the membrane will be permeabilised or not, which will be followed by outer membrane permeabilisation and release of cytochrome c. The outer membrane permeabilisation occurs via the mitochondrial apoptosis-induced channel (MAC) which is suggested to interact and activate the mPTP (in the inner membrane) via positive reinforcement loops, leading to matrix swelling, loss of folding and cristae, rupture of the inner membrane and more cytochrome c release (Kinnally et al., 2011). Other outer membrane channels, such as VDAC1, have also been implicated in the facilitation of the mPTP opening, due to an increased Ca^2+^ influx and activation of the calcium-sensitive mPTP. VDACs are also under the influence of the Bcl-2 protein family (Heiden et al., 2001).

Few studies have investigated the role of mitochondria in apoptosis and wound healing. Herein, a study in *C. elegans* wounding model has suggested a possible role of mPTP in wound healing (Xu and Chisholm, 2014). Xu et al. showed that mitochondrial calcium induces mtROS upon injury, which is then released via the mPTP and accelerates wound healing. As such, further studies are required to further elucidate the role of mPTP in wound healing. Additionally, investigations into other mechanisms mentioned in this review such as the metabolism of apoptosis, as well as the interaction of mitochondrial dynamics and mitophagy with apoptosis in the context of wound healing are required.

## 6 Mitochondrial dynamics and mitophagy

**FIGURE 4:**
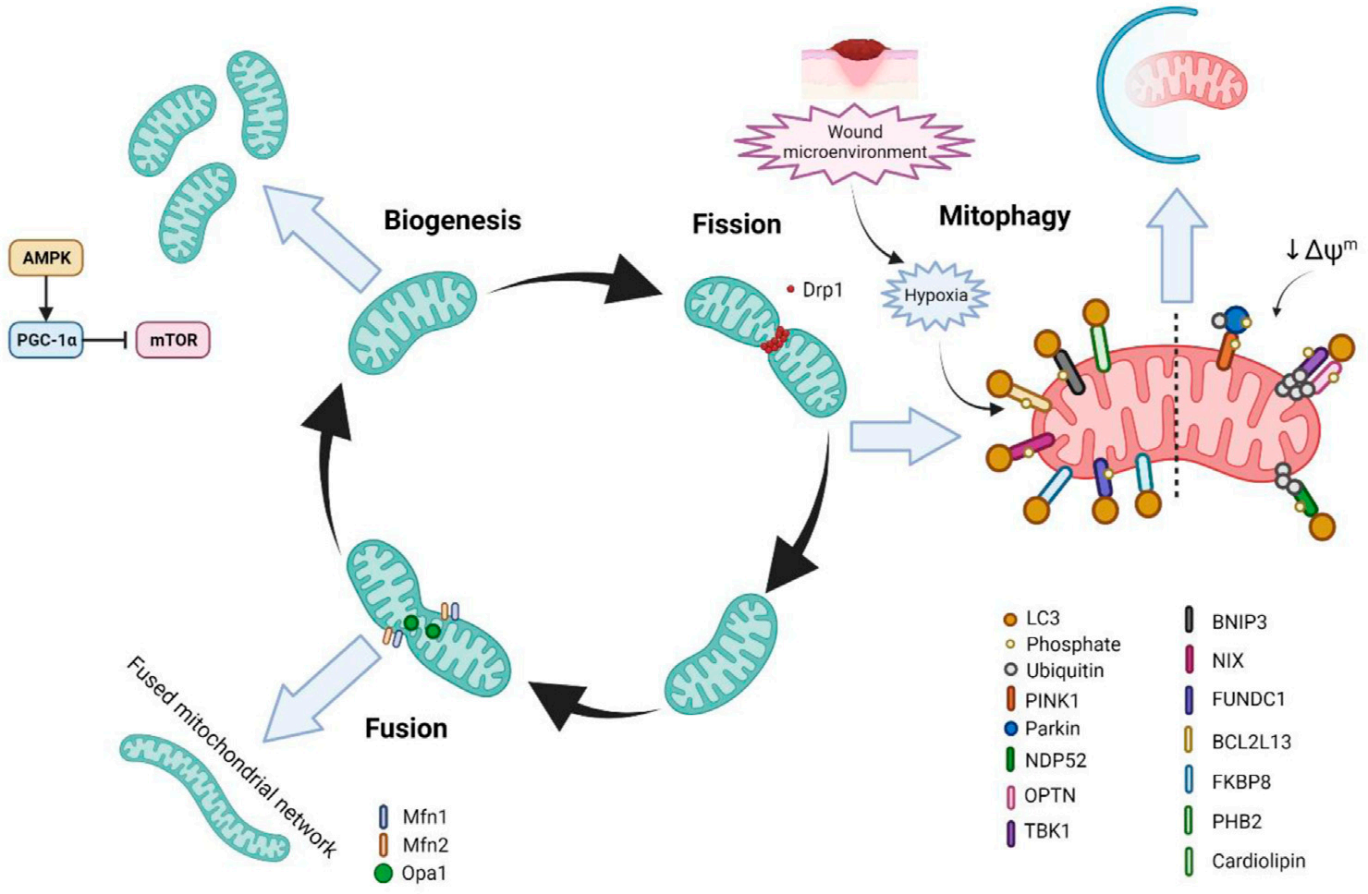
The life cycle of mitochondria and mitochondrial homeostasis and wound healing. In response to increased energetic demand in the cell, mitochondria can fuse with other mitochondria as well as increase their number through mitochondrial biogenesis (mitogenesis). In contrast, when the energy requirements in the cell are diminished or the cell is stressed, mitochondria can undergo fission/fragmentation in order to reprogramme the cellular metabolic state. Further, mitochondria can undergo degradation through mitophagy in order to clear damaged mitochondria and to prevent cellular stress.

Mitochondria can rapidly adapt in order to meet changing metabolic demands in the cell or respond to cell stressors. In general, greater demand is met with increased mitochondrial biogenesis (mitogenesis) as well as fusion of mitochondria in order to expand the network. In contrast, decreased demand or certain stressors induce the fission of mitochondria and in some cases degradation through mitochondrial autophagy (mitophagy) (Herst et al., 2017; Ma et al., 2020) (Figure 4).

### 6.1 Mitochondrial dynamics

Mitochondria undergo constant cycles of fission and fusion events in what is termed ‘mitochondrial dynamics’. Mitochondrial dynamics control the morphology of mitochondria, as well as the number, size, distribution, transport, and quality control (Chen and Chan, 2009). Correct maintenance of these factors is important in order for the regulation of mitochondrial function. Over the past decades evidence has supported the fact that mitochondrial dynamics is important for several cellular processes, such as proliferation, cell-cycle progression, Ca^2+^ homeostasis, and cell death, and that dysregulation in mitochondrial dynamics is associated with several diseases (Roy et al., 2015; Chen and Chan, 2017; Trotta and Chipuk, 2017; Altieri, 2018). Dynamin-related protein 1 (Drp1) is the master regulator of mitochondrial fission and is recruited to the outer mitochondrial membrane (OMM) by its receptors Fis1, Mff, MIEF1, and MIEF2. Once recruited, Drp1 wraps around the mitochondria and induces constriction through GTPase activity (Otera et al., 2013). With regards to mitochondrial fusion, there are three key proteins–Opa1, Mfn1, and Mfn2. Mfn1 and Mfn2 lie on the OMM and are responsible for forming a dock between two adjacent mitochondria followed by fusion through GTP hydrolysis (Brandt et al., 2016). In addition to its fusion functions, Mfn2 is also involved in insulin signalling, energy metabolism, ER-mitochondrial connections, and mitophagy (de Brito and Scorrano, 2008; Perumalsamy et al., 2010; Chen and Dorn, 2013; Zorzano et al., 2015). Opa1 on the other hand is located in the IMM, and is responsible for the fusion of the IMM and matrixes of two adjacent mitochondria (Ishihara et al., 2006; Mishra et al., 2014).

In recent years, studies have shown that mitochondrial fission and therefore a fragmented mitochondrial phenotype, where the mitochondria are more spherical and unfused, and the mitochondrial network is less tubular and branched, is important for wound healing (Muliyil et al., 2011; Muliyil and Narasimha, 2014; Fu et al., 2020; Ponte et al., 2020). In the Ponte *et al.* study, in which wound healing was characterised in *Drosophila* with various mutations in mitochondrial dynamic genes, they found that Drp1-mediated fission and an increased fragmented phenotype regulated wound healing by controlling ROS production, Ca^2+^ homeostasis, and the accumulation of F-actin at the wound edge (Ponte et al., 2020). In this study it was also found that mutations in Fis1 leading to mitochondrial fragmentation also impaired wound healing. Similarly, in the Fu et al. study it was shown that mitochondrial fission is important in wound healing in *C.elegans* and zebrafish, however in this study they found that mitochondrial fragmentation that occurs immediately after wounding was Drp1-independent, and instead dependant on the Rho GTPase MIRO-1 (Fu et al., 2020). It was also demonstrated that this mitochondrial fragmentation-mediated acceleration of wound healing relied on mtROS production and Ca^2+^ signalling, as well as being associated with a more glycolytic metabolism (Fu et al., 2020). It is important to note however that neither of these studies looked at the role of mitochondrial dynamics in human tissue, which may be a factor in the differences in findings. In addition, few of these studies used mouse models, which although considerable differences exist between wound healing in human and mice, such as the significant presence of *panniculus carnosus* (Gerber et al., 2014) and stem cells niches from increased hair follicle frequency in mice (Eming et al., 2014), these models offer an important opportunity to study wound healing pathophysiology at a higher organismal level (Zomer and Trentin, 2018), and also strongly supports the notion that studies into human tissue are required. Importantly, a lack of electron microscopy analysis of the ultrastructure of mitochondrial morphology in these studies limits the knowledge of cristae structure and complexity in wound healing contexts.

Previous studies have shown that mitochondria in differentiating epidermal keratinocytes have a fragmented phenotype (Mellem et al., 2017; Ipponjima et al., 2020). This mitochondrial fragmentation coincides with the reduced energetic requirement of the keratinocytes involved in differentiation, as opposed to proliferating basal keratinocytes, and aids in the degradation of mitochondria required in keratinocyte differentiation, as discussed in the Section 6.3 later.

Mitochondrial fragmentation is known to promote apoptosis of macrophages (Wang et al., 2017), cell proliferation (Parker et al., 2015), and support glucose homeostasis and glycolytic metabolism (Jakobs et al., 2003; Toda et al., 2016; Seo et al., 2018) in a variety of tissues. Although the role of mitochondrial fragmentation in these processes has not been studied in the wound healing context in particular, appropriate regulation of these physiological processes is known to be critical to well-coordinated wound healing.

### 6.2 Mitochondrial biogenesis

Mitogenesis is commonly initiated in cases of decreased ATP levels, specifically through the AMPK-peroxisome proliferator-activated receptor γ coactivator 1α (PGC-1α) pathway. PGC-1α subsequently interacts with nuclear respiratory factor 1 (NRF1) to activate the mitochondrial transcription factor A (TFAM) and induce transcription of mtDNA (Chung et al., 2017). The AMPK-PGC1α pathway also inhibits mammalian target of rapamycin (mTOR) signalling to decrease anabolic processes and promote oxidative metabolism (Arnould et al., 2015).

The role of mitogenesis in wound healing has not been extensively investigated, and so requires further study. However, it has been examined in some studies, and from current knowledge of mitochondrial homeostasis it can be suggested that decreased mitogenesis is seen in the early stages of wound healing where glycolysis is the main source of metabolism. One such previous report that has investigated mitochondrial homeostasis in wound healing found that macrophages involved in the later stages of wound healing had higher mitochondrial mass, as determined by MitoTracker Green staining, as well as a transcriptional upregulation of genes involved in mtDNA replication and biogenesis, when compared to macrophages involved in the inflammatory phase (Willenborg et al., 2020). Conversely though, higher mitochondrial mass was observed in dermal fibroblasts in day 1, 7, and CW patient tissue in another study (Manchanda et al., 2023).

Increased mitogenesis has been described in other tissues such as in rotator cuff tendon injury muscle healing (Thankam et al., 2018). However, in this tissue mitogenesis and OXPHOS are upregulated in response to hypoxia, which is seemingly the opposite of what happens in the skin wound healing context.

### 6.3 Mitophagy

Mitophagy is the selective clearance of mitochondria by autophagy, and effective regulation of mitophagy is essential for the functional integrity of the mitochondrial network and cell homeostasis (Levine and Kroemer, 2008; Ma et al., 2020). Indeed, defective mitophagy and the subsequent accumulation of dysfunctional mitochondria is implicated in several diseases such as Parkinson’s, neurodegenerative diseases, and type 2 diabetes, among others (Doblado et al., 2021). Numerous pathways of mitophagy have been described, such as PINK1/Parkin ubiquitin-dependant mitophagy, as well as receptor-dependent pathways, which include BNIP3L/NIX (herein referred to as NIX), BNIP3, and FUNDC1, which interact with LC3 to induce mitochondrial degradation (Ma et al., 2020).

Mitophagy is known to promote cell proliferation by helping switch metabolism from OXPHOS toward aerobic glycolysis (Doblado et al., 2021). Mitophagy has also been shown to drive metabolism towards glycolysis in order to promote cell growth and survival in cancer cells (Domenech et al., 2015; Esteban-Martinez et al., 2015; Agnihotri et al., 2016), as well as the metabolic reprogramming required during somatic stem cell differentiation. Additionally, it is known that HIF-1α induces the transcription of mitophagy receptors BNIP3, NIX, and FUNDC1 (Sowter et al., 2001; Esteban-Martínez et al., 2017). Although the role of mitophagy in metabolism in wound healing has not been investigated, current thinking supports the idea that it may play a critical role (Liu et al., 2020).

Various studies have investigated the role of mitophagy in different aspects of wound healing, however, few studies have undertaken studies using human wounded tissue. One such aspect that has been studied is the migration of epidermal cells during re-epithelialisation. Here, previous studies in mice have demonstrated the role of BNIP3-mediated mitophagy in accelerating keratinocyte migration, and that this upregulation of BNIP3 was controlled by hypoxia (Zhang et al., 2017; Zhang et al., 2019). Another recent study found that hypoxia-induced mitophagy promoted keratinocyte migration and proliferation by degrading p-MAP4 (Feng et al., 2023). Although not investigated in the context of wound healing, mitophagy and autophagy in general are known to regulate the acquisition of mesenchymal markers, focal adhesion disassembly, maintenance of the cytoskeleton organisation as well as focal adhesion, and β1 integrin membrane recycling to promote cell migration (Tuloup-Minguez et al., 2013; Maes et al., 2014; Sharifi et al., 2016; Liu et al., 2017; Sun et al., 2018). Interestingly, autophagy has been shown to be important epithelial-to-mesenchymal (EMT) transition in some cancers, although the molecular mechanism behind this is not fully known (Gugnoni et al., 2016).

Significantly, BNIP3 (Moriyama et al., 2014; Moriyama et al., 2017) and NIX (Simpson et al., 2021) mediated mitophagy has been shown to be essential for the differentiation of keratinocytes in the epidermis. General autophagy has also been demonstrated to play a role in epidermal keratinocyte differentiation (Aymard et al., 2010; Chatterjea et al., 2011; Yoshihara et al., 2015; Akinduro et al., 2016; Sil et al., 2018; Ipponjima et al., 2020). However, studies by Moriyama *et al.* and the Simpson *et al.* study were the first to specifically describe mitophagy (Moriyama et al., 2014; Moriyama et al., 2017; Simpson et al., 2021). Additionally, they showed that the mitochondria in differentiating keratinocytes were more depolarised, had higher levels of HIF-1α expression, upregulated Drp1-mediated fragmentation, and were using glycolysis for metabolism–linking previously discussed aspects of mitochondrial homeostasis. Importantly, through inhibition of these functions, the keratinocytes displayed abnormal differentiation. This suggests that dysfunctional mitophagy could be implicated in abnormal re-epithelialisation and therefore in the pathogenesis of chronic wounds (Simpson et al., 2021).

Previous studies have shown that BNIP3 mitophagy protects against UVA (Zhao et al., 2013) and UVB-induced as well as ROS-mediated epidermal damage by preventing excessive apoptosis (Moriyama et al., 2014; Moriyama et al., 2017). Additionally, clinical trials in which the mitophagy inducer urolithin A (UA) was applied topically demonstrated beneficial effects of mitophagy on skin ageing and protection from UVB-induced photodamage (D’Amico et al., 2023; Liu et al., 2022). One potential mechanism could be that mitophagy aids in preventing the accumulation of damaged and oxidised proteins, thereby supressing proteolytic stress (Ogata et al., 2006; Mera et al., 2010; Song et al., 2017). Although not confirmed in studies, this would suggest that functioning mitophagy may be important in preventing chronic wound pathogenesis by inhibiting excessive apoptosis and the pathophysiological issues that arise with that (Ogata et al., 2006; Mera et al., 2010; Burton et al., 2013).

PINK1 has been shown to be important to the function of astrocytes in neurological wound healing (Choi et al., 2013), however, through screenings of altered signalling pathways, PINK1/Parkin mediated mitophagy has been shown to not play an important role in skin wound healing. This suggests that the receptor-dependent pathways of mitophagy, such as the BNIP3, NIX, and FUNDC1 pathways, as opposed to the PINK1/Parkin pathway, are involved in wound healing. This agrees with the fact that receptor-dependant pathways are known to be upregulated by hypoxia-induced HIF-1α activation (Zhang and Ney, 2009; Liu et al., 2012). However, future studies would benefit from utilising mouse models with knockouts of mitophagy receptors.

With regards to mitophagy in inflammation, although it has not been studied in the wound healing context, mitophagy has been shown to prevent the activation of the inflammasome by clearing damaged mitochondria and thereby preventing the excess release of DAMPs (Lupfer et al., 2013; Guo et al., 2014; West et al., 2015; Kim et al., 2016; Zhong et al., 2016). In addition, mitophagy contributes to the metabolic rewiring of macrophages in order to stimulate either M1 or M2 macrophage functions (Wu et al., 2014; Gupta et al., 2019; Lampert et al., 2019). It is feasible therefore that mitophagy plays an important role in the inflammatory stage of wound healing, and as such, dysregulated mitophagy may contribute to excess inflammation and the pathogenesis of chronic wounds.

Finally, another recent study found that pharmacological induction of mitophagy promoted angiogenesis and the deposition of mature collagen fibres to enhance wound healing (Feng et al., 2022).

## 7 Other functions of mitochondria

### 7.1 Mitochondria, calcium, and wound healing

Ca^2+^ plays an essential role in wound healing, and mitochondria are one of the key regulators of Ca^2+^ signalling in cells. However, few studies have specifically investigated the relationship of mitochondria and Ca^2+^ in wound healing.

The elevation of intracellular Ca^2+^ is one of the first damage signals in the wound healing process (Lansdown, 2002) and is essential for the initiation and regulation of wound healing (Xu and Chisholm, 2011; Yoo et al., 2011; Razzell et al., 2013; Xu and Chisholm, 2014). As mentioned previously in this review, this propagation of Ca^2+^ is responsible for various processes such as being inflammatory mediators (Niethammer et al., 2009a; Niethammer et al., 2009b; Razzell et al., 2013) and modulating proliferation, migration, and differentiation of fibroblasts and keratinocytes during re-epithelialisation (Hennings and Holbrook, 1983; Ko et al., 2001; Huang et al., 2006; Bikle et al., 2012; Pastar et al., 2014; Xu and Chisholm, 2014; Law et al., 2015; Xue and Jackson, 2015). Ca^2+^ is also involved in angiogenesis in wound healing (Berridge et al., 1998), as well as triggering an increased generation and release of mtROS (Xu and Chisholm, 2011; Xu and Chisholm, 2014). Additionally, Ca^2+^ signalling pathways contribute to the regulation of metabolism and extracellular matrix formation (Subramaniam et al., 2021).

As stated above, mitochondria are significantly responsible for regulating cellular Ca^2+^ storage and propagation, and mitochondrial Ca^2+^ is essential for various cellular signalling pathways as well as in cellular bioenergetics (Pathak and Trebak, 2018). Ca^2+^ is imported into the mitochondria through the mitochondrial Ca^2+^ uniporter (MCU) and effluxed via the mitochondrial Na^+^/Ca^2+^/Li^+^ exchanger (NCLX). However, because mitochondria have a low affinity for Ca^2+^, Ca^2+^ transfer into the mitochondria occurs through close contacts with the ER, termed mitochondrial associated membranes (MEM) (Mesmin, 2016). These MEMs are essential for various mitochondrial functions, such as glucose sensing, mitophagy, apoptosis, the unfolded protein response (UPR), insulin signalling, and ROS signalling (Rieusset, 2018). Recent studies have described the important role of Ca^2+^ in regulating oxidative stress, as well as mitochondrial dynamics and biogenesis, in aiding wound healing, particularly re-epithelialisation (Xu and Chisholm, 2011; Xu and Chisholm, 2014; Hunter et al., 2018; Ponte et al., 2020). In addition, a study by Parnis et al. demonstrated that mitochondrial calcium homeostasis was important in cell proliferation and wound healing in astrocytes (Parnis et al., 2013). However, much is still unknown about the roles of mitochondrial Ca^2+^ homeostasis in skin wound healing, such as its role in apoptosis and immune responses, particularly in humans.

### 7.2 Mitochondrial impact on inflammation

Mitochondria play an essential role in the regulation of immune cells, primarily through acting as hubs for metabolism regulation, which is necessary for the activation of several immune cell types of both the innate and acquired immune systems. In addition, they participate in various inflammatory signalling pathways via ROS mediators (Chandel et al., 2000; Gurung et al., 2015), can form scaffolding required for interactions between various immune cells and respective proteins (Jacobs and Coyne, 2013), and components of mitochondria can activate several types of inflammatory responses themselves (Marchi et al., 2023).

In general, inflammation is initiated by the activation of pattern recognition receptors (PRRs), which can be achieved by bacterial and viral molecules, as well as endogenous DAMPs (Kroemer et al., 2022). DAMPs are normally restricted physical access to PRRs in physiological conditions, however, in various cellular stress environments whereby alterations in the permeability of cell compartments occurs, DAMPs can interact with PRRs and initiate immune responses (Elliott et al., 2009; Ghiringhelli et al., 2009). Notably, mitochondria contain DAMPs (mtDAMPs) which can activate immune responses in different tissues. A notable example which has gathered significant interest in recent years is the role of mtDNA in the cyclic GMP-AMP synthase (cGAS), stimulator of interferon response cGAMP interactor 1 (STING1) pathway (Decout et al., 2021). cGAS is a cytosolic protein that responds to dsDNA in the cytosol by catalysing the formation of cyclic GMP-AMP (cGAMP), which then binds to ER-bound STING1 to initiate interferon responses (Decout et al., 2021). Subsequently, studies in recent years have shown that mtDNA in the cytosol is also a potent activator of the cGAS-STING pathway, and has been shown to contribute to various diseases, such as systemic lupus erythematosus, liver damage, and ageing itself (West et al., 2015; Kim et al., 2019; Miller et al., 2021; He et al., 2022). There have been numerous methods proposed to as to how mtDNA is released into the cytosol, including following mitochondrial outer membrane permalisation (MOMP) (Fuchs and Steller, 2015; Li et al., 2021), which involves the BAX-BAK1 pores (McArthur et al., 2018; Riley et al., 2018; Yamazaki et al., 2020; Willemsen et al., 2021), or BH3-interacting domain death agonist (BID)-induced mitochondrial membrane pores (Flores-Romero et al., 2022). Additionally, cytosolic mtDNA translocation has also been shown to occur without significant mitochondrial stress through MPT responses via VDAC pores (Kim et al., 2019; Yu et al., 2020; Domizio et al., 2022). As well as being activators of the cGAS-STING1 pathway, mtDNA can also activate inflammasome signalling, in particular NLRP3 and AIM2 containing inflammasomes (Shimada et al., 2012; Dang et al., 2017). mtDNA involved in these inflammasome pathways are generally oxidised, released via MPT (Nakahira et al., 2011; Renaudin, 2021), and can initiate a feed-forward loop whereby increased ROS further increased mtDNA release into the cytosol. As well as the inflammatory pathways mentioned above, mtDNA can also activate Toll-like receptor 9 (TLR9) and advanced glycosylation end product-specific receptors (AGER/RAGE) to subsequently activate myeloid cells (Vanpouille-Box et al., 2019).

Another mtDAMP is mtRNA, which has been shown to activate RIG-1 like receptors (Hur, 2019). As with mtDNA release into the cytosol, mtRNA can be released via BAX-BAK1 induced MOMP (Dhir et al., 2018). Other components of mitochondria are also known to induce various forms of inflammation. These include SMAC, which is released downstream of MOMP and can modulate T cells (Gyrd-Hansen and Meier, 2010; Rizk et al., 2019), *N*-formyl peptides and cardiolipin, which activate neutrophils (Boada-Romero et al., 2020) and T cells (Leslie et al., 2008; Dieudé et al., 2011) respectively, as well as cytochrome *c,* which has been shown to stimulate TLR4 inflammation (Gouveia et al., 2017).

Several of the immune systems mentioned above are essential in the wound healing process, including TLR9-mediated inflammation (Yamamoto et al., 2011) and inflammasomes (Ito et al., 2018). In addition, the interferon response plays an important role in wound healing, in particular through antimicrobial activities in early wound healing, and by promoting re-epithelialisation (Decker et al., 2005; Stetson and Medzhitov, 2006). In a recent study on mice topically treated with cGAMP, the cGAS-STING1 pathway was shown to accelerate wound closure, in particular by increasing cell migration to the wound site (Mizutani et al., 2020). Importantly, treatment with IFNR inhibitors also significantly impaired wound healing in these mice. Although few studies have looked at the specific role of mitochondria in these immune responses in the wound healing process, current knowledge supports the theory that mitochondria are involved. This suggests that future studies should look to elucidate the potential contribution of mitochondria to the activation of immune pathways in wound healing.

With regards to the role of mitochondrial metabolism in immune signalling, increased glycolysis at the early stages and a shift towards more oxidative metabolism towards the later wound healing stages has been shown to control macrophage phenotype and polarisation in wound healing, as discussed previously (Willenborg et al., 2020). Mitochondrial metabolism is also essential for the proper function of neutrophils. In particular, they play a role in antimicrobial activities through respiratory burst, as well as the development, adhesion, migration, and ultimately death of neutrophils (Cao et al., 2022). For example, pharmacological inhibition of OXPHOS reduced neutrophil ROS production to decrease the potency of their respiratory burst (Bao et al., 2014; Dunham-Snary et al., 2022). Similar pharmacological OXPHOS inhibition also reduced neutrophil migration and chemotaxis (Chen et al., 2006; Bao et al., 2015). Importantly however, too much ATP production also reduced chemotaxis in neutrophils (Kondo et al., 2019), suggesting that there is a well-regulated balance in the regulation of metabolism required. Finally, mitochondrial metabolism is required for the induction of apoptosis of another important immune cell type in wound healing, platelets (Melchinger et al., 2019). However, apart from the examples listed, few studies have investigated the role of mitochondrial metabolism in immune cells in the context of wound healing.

In summary, even though there is a large body of literature describing the importance of inflammation in wound healing as well as that of mitochondria in inflammation, little is known about the specific role of mitochondria in inflammation in wound healing. Therefore, future studies should look to bridge the gap in scientific knowledge in this area.

### 7.3 Mitochondrial unfolded protein response

Another aspect of mitochondrial quality control is the mitochondrial unfolded protein response (UPR^mt^). Like mitophagy, the UPR^mt^ is responsible for orchestrating responses to mitochondrial stresses such as the aggregation of misfolded mitochondrial proteins or mutated mtDNA (Zhao et al., 2002), although unlike mitophagy, the UPR^mt^ does not result in the degradation of damaged mitochondria, but instead, promotes the repair of individual mitochondria and the mitochondrial network as a whole (Shpilka and Haynes, 2018). The UPR^mt^ is also important in the context of mitochondria in ageing (Lima et al., 2022). Mechanistically, the UPR^mt^ involves the activation of the nuclear localising sequence (NLS) of the activating transcription factor associated with stress (ATFS-1). In a healthy mitochondrial network context, the mitochondrial localising sequence (MLS), predominates over the NLS, and directs ATFS-1 towards import into the mitochondria, where it is degraded by the protease LON (Nargund et al., 2012; Rauthan et al., 2013). However, in the event of mitochondrial stress, such as an accumulation of OXPHOS complexes (Houtkooper et al., 2013), ATFS-1 is activated as a nuclear transcription factor and leads to the subsequent activation of the UPR^mt^, in order to avoid toxicity (Wang and Chen, 2015; Wrobel et al., 2015).

The UPR^mt^ is similar to the ER-directed UPR (herein referred to simply as UPR), in that they contain several of the same proteases and chaperones, including CHOP, and both the UPR and UPR^mt^ reduce cellular damage by clearing misfolded proteins (Hetz, 2012). Additionally, the UPR is significantly involved in the induction of apoptosis, cell differentiation, inflammatory responses, and glucose and lipid metabolism–all of which are important in the context of mitochondria in wound healing (Hetz, 2012). Indeed, mild UPR has been shown to be beneficial to wound healing, however, excessive UPR induces cell death and can be detrimental to wound healing (Bachar-Wikstrom et al., 2021). Currently, little is known about the role of the UPR^mt^ in wound healing or skin ageing in general. Therefore, future studies should aim to investigate its role, as well as the potential link between the UPR and UPR^mt^ in the context of wound healing.

## 8 Conclusion and future perspectives

**FIGURE 5:**
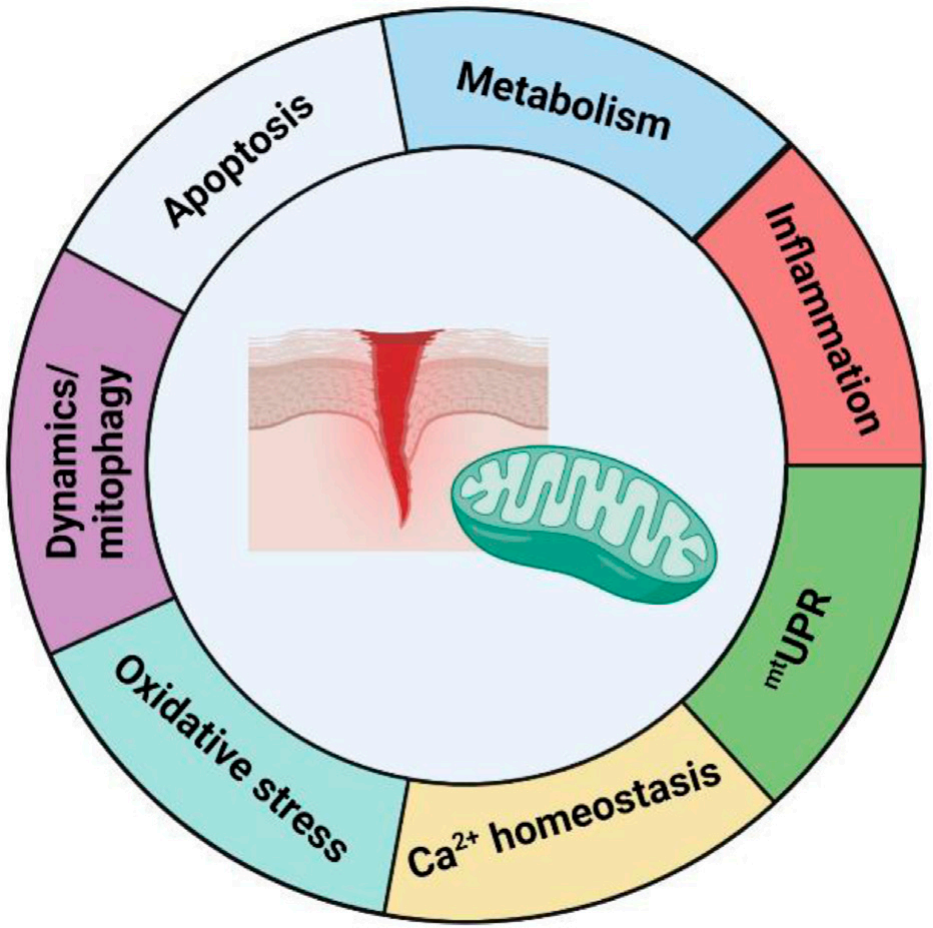
Summary of the role of mitochondria in wound healing. Current knowledge about the role of mitochondria in wound healing can be divided into seven sections–mitochondrial metabolism, oxidative stress/ROS signalling, mitochondrial dynamics and mitophagy, apoptosis, inflammation, ^mt^UPR, and Ca^2+^ homeostasis. Although a considerable amount of knowledge has been learned in the past decade, many questions remain about its role, and further research is needed in order to develop translational options for the treatment of mitochondrial parameters in chronic wounds.

In recent years, mitochondria have become the most studied organelle in biomedical research (Picard and Shirihai, 2022; Monzel et al., 2023), and significant research has collectively demonstrated that mitochondria play a vital role in various aspects of the wound healing process. Indeed, dysfunction in numerous aspects of mitochondrial behaviours and functions is significantly implicated in the pathogenesis of chronic wounds. Additionally, although wound healing has not been noted in many cases of mitochondrial disease patients, approximately 10% of these patients present with some form of skin pathology (Hussain et al., 2021). This review has highlighted the several ways in which mitochondria are involved in wound healing, notably by regulating cellular metabolism, as well as redox signalling and apoptosis, which are critical mediators of correct wound healing function (Figure 5). In addition, we have discussed how control of mitochondrial homeostasis through the regulation of mitochondrial dynamics, mitogenesis, and mitophagy are also important in wound healing.

However though, one of the significant limitations of research in the field of mitochondria in wound healing, and of wound healing as a whole, is the fact that few studies use human tissue. This can limit the translational potential of many findings, as human skin and various factors of the wound healing process itself varies considerably to that of animal models such as mice, *Drosophila*, or *C.elegans*. These studies are still important in furthering knowledge in physiological and pathological mechanisms of wound healing, however future studies should aim to also use human tissue when studying mitochondria in wound healing (Zomer and Trentin, 2018). It would be hoped that in the near future more studies can look to target various aspects of mitochondrial function in order to explore the therapeutic potential of targeting mitochondria in chronic wounds. Such targeting may include promoting glycolysis for improved haemostasis and inflammation stage function, ROS dynamics, and ensuring proper mitochondrial dynamics, particularly in the case of elderly individuals. With regard to targeting mitochondrial metabolism, several chemicals that inhibit specific forms of mitochondrial metabolism, such as metformin, which inhibits the ETC, are available and undergoing clinical trials for various diseases including some forms of cancer (Vasan et al., 2020; Sainero-Alcolado et al., 2022). There are also numerous ways to modulate mtROS levels therapeutically, including by inhibiting the ETC (Perillo et al., 2020), or the glutathione disulphide mimetic NOV-002, which has also been used in clinical trails for various cancers (Montero and Jassem, 2011). Finally, one exciting, although yet not fully validated, potential treatment of mitochondrial defect-related pathology is that of mitochondrial transplantation (Ulger and Kubat, 2022). Broadly, there are several alternative *in vitro* methodologies for this application including microinjection (Capecchi, 1980), magnetomitotransfer (Macheiner et al., 2016), the photothermal nanoblade method (Wu et al., 2011), or direct transfer into tissue in an *in vivo* manner. With regards to the latter method, early studies have shown beneficial impacts of directly transferring mitochondria into mouse models of ischemic heart disease (McCully et al., 2009), liver ischemia-reperfusion (Lin et al., 2013), ischemic spinal chords (Fang et al., 2021), and Parkinson’s disease (Shi et al., 2017). As such, due to the partial overlap in pathophysiological factors of these diseases and chronic wounds, for example, chronic inflammation and oxidative stress, as well as metabolic imbalances, the application of mitochondrial transplantation should be considered as a potential future treatment option.

Importantly though, as the pathogenesis and pathophysiology of chronic wounds can vary greatly between patients as a result of differences in genetic backgrounds, chronic wound type, or lifestyle factors (Falanga et al., 2022), future therapeutic targeting should look at how any rational therapy specifically impacts mitochondrial function, with the aim for specific and targeted benefits. This type of personalised medicine is essential in treating cancer, and this approach should be adopted for treating chronic wounds. For instance, a treatment targeted at increasing glycolytic metabolism with the aim of improving immune cell function should be assessed to see whether this can have harmful off-target effects, and the specific duration and timeline of the treatment should be determined. With regard to that, advances in emerging technologies such as combining metabolomics and proteomics on human wound samples at various stages of the wound healing cycle could provide hints to how active metabolic pathways vary at different time points (Karczewski and Snyder, 2018). In addition, single cell studies such as single-cell proteomics or deep visual proteomics (DVP) on human tissue may provide insights into the relationship of specific cells types such as fibroblasts or ECM cells and arrays of mitochondrial parameters (Rosenberger et al., 2023).
